# Eco-friendly synthesized nanoparticles as antimicrobial agents: an updated review

**DOI:** 10.3389/fcimb.2023.1224778

**Published:** 2023-08-16

**Authors:** Shilpa Borehalli Mayegowda, Arpita Roy, Manjula N. G., Soumya Pandit, Saad Alghamdi, Mazen Almehmadi, Mamdouh Allahyani, Nasser S. Awwad, Rohit Sharma

**Affiliations:** ^1^ Department of Psychology, CHRIST (Deemed to be University), Bangalore, India; ^2^ Department of Biotechnology, School of Engineering & Technology, Sharda University, Greater Noida, India; ^3^ Department of Microbiology, School of Basic and Applied Sciences, Dayananda Sagar University, Bengaluru, India; ^4^ Department of Life Sciences, School of Basic Science and Research, Sharda University, Greater Noida, India; ^5^ Department of Biotechnology, Graphic Era Deemed to be University, Dehradun, Uttarakhand, India; ^6^ Laboratory Medicine Department, Faculty of Applied Medical Sciences, Umm Al-Qura University, Makkah, Saudi Arabia; ^7^ Department of Clinical Laboratory Sciences, College of Applied Medical Sciences, Taif University, Taif, Saudi Arabia; ^8^ Department of Chemistry, King Khalid University, Abha, Saudi Arabia; ^9^ Department of Rasa Shastra and Bhaishajya Kalpana, Faculty of Ayurveda, Institute of Medical Sciences, Banaras Hindu University, Varanasi, India

**Keywords:** green synthesis, antimicrobial agents, anticancer agents, antioxidant activity, drug delivery, DNA damage, eco-friendly

## Abstract

Green synthesis of NPs has gained extensive acceptance as they are reliable, eco-friendly, sustainable, and stable. Chemically synthesized NPs cause lung inflammation, heart problems, liver dysfunction, immune suppression, organ accumulation, and altered metabolism, leading to organ-specific toxicity. NPs synthesized from plants and microbes are biologically safe and cost-effective. These microbes and plant sources can consume and accumulate inorganic metal ions from their adjacent niches, thus synthesizing extracellular and intracellular NPs. These inherent characteristics of biological cells to process and modify inorganic metal ions into NPs have helped explore an area of biochemical analysis. Biological entities or their extracts used in NPs include algae, bacteria, fungi, actinomycetes, viruses, yeasts, and plants, with varying capabilities through the bioreduction of metallic NPs. These biosynthesized NPs have a wide range of pharmaceutical applications, such as tissue engineering, detection of pathogens or proteins, antimicrobial agents, anticancer mediators, vehicles for drug delivery, formulations for functional foods, and identification of pathogens, which can contribute to translational research in medical applications. NPs have various applications in the food and drug packaging industry, agriculture, and environmental remediation.

## Introduction

**Figure 1:**
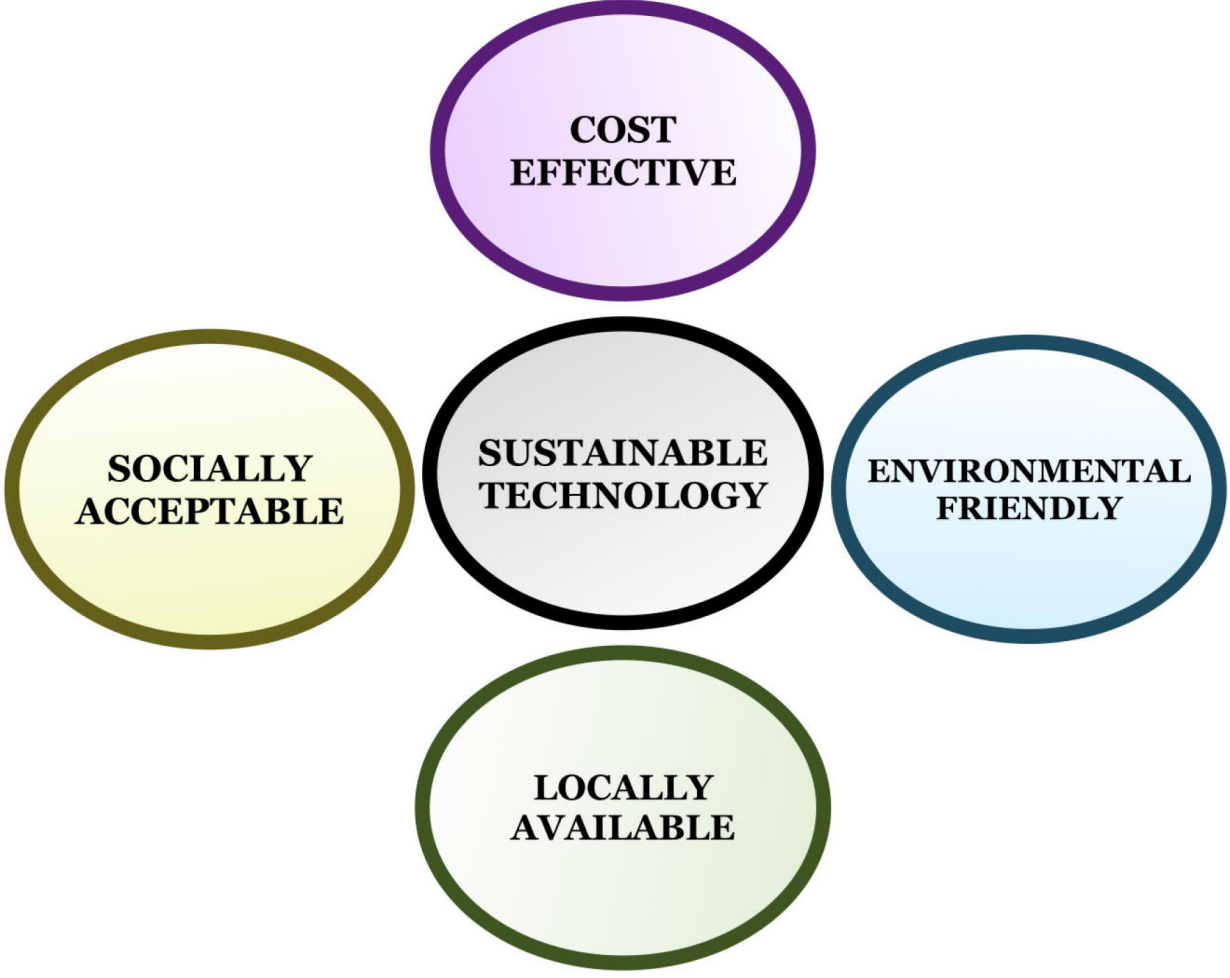
Implication of green nanotechnology.

Nanoparticles (NP) vary in size from 1 to 100 nm with a large surface-to-volume ratio and are currently used in translation research technology. The biological NPs employed are ecofriendly, cost effective, and have attracted substantial attention. Nanotechnology comprises various fields, including biological sciences, chemistry, physics, material science, engineering, and computational science. It has applications in various fields, such as optics, space industries, cosmetics, biomedical, chemical industries, electronics, mechanics, environmental remediation, food and feed, health care, photoelectron chemistry, numerous engineering fields, and material science (Christian et al., 2008). Nanotechnology is the most promising field in integrated engineering, physics, medicine, chemistry, and biology, and has widespread applications with increased demand for industrial-scale production of nanomaterials (NMs). However, concerns have been raised regarding the environmental safety of chemically synthesized NPs. The origin and synthesis of these chemical NPs have caused an enormous burden on the environment because of their toxins, which have harmful effects on animals and humans (Wiley et al., 2005; Vo-Dinh, 2007). Physiochemical techniques to produce NPs of metals and metal oxides require the use of corrosive and toxic reducing agents such as sodium borohydride and hydrazine hydrate, which adversely affect the atmosphere (Mandal et al., 2006). Thus, alternative methods for synthesizing harmless NPs using moderate solvents are eco-friendly reducers and stabilizers with experimental parameters or biological material applications. The most eco-friendly are the use of plants, fungal or bacterial extracts, lysates, or biomolecules, which are widely used due to their minimal side effects (**Figure 1**). Numerous microbes act as potential sustainable precursors that are eco-friendly and can produce stable and well-functionalized NPs (Baroumand Moghaddam et al., 2015). Thus, such environmentally safe techniques used for the synthesis of NPs are also referred to as ‘green nanotechnology’ or “clean-technology” that are feasible alternatives to chemical methods. Furthermore, nanoparticles conjugated with natural biomolecules exhibit improved bioavailability and minimal side effects. They are not only smaller in size with higher permeability, but are also important reducing and stabilizing agents and show excellent antioxidant activity. Nanoparticles serve as potential antimicrobial agents because of their affinity toward sulfur-rich amino acids, adherence to the microbial cell wall by electrostatic attraction, and disruption of the microbial cytoplasmic membrane and nucleic acids. They possess anticancer activity owing to the initiation of oxidative stress, cellular DNA damage, and lipid peroxidation. Additionally, biological synthesis is eco-friendly, cost-effective, and fast, and unlike compounds or their derivatives, it is not harmful, thus minimizing pollution. Therefore, the use of green synthetic methods holds promise as safe alternatives for healthcare and bioremediation applications. Currently, nanotechnology, being an indispensable part of the healthcare, research and innovation sector in recent years is used extensively (Manjula et al., 2022). Nanocompounds have been promising antimicrobial agents, anti-cancer mediators, vehicles for drug delivery, formulations for functional foods, in the identification of pathogens, and in the food and drug packaging industry.

## Sustainable green nanotechnology

### Advantage

Engineered nanomaterials (NMs) have gained the utmost importance in daily life and have been used in cosmetics, food packaging materials, biosensors, drug delivery systems, and therapies. Subsequently, their size is similar to that of biological macromolecules with antibacterial and anti-odor properties, which can be widely used in wound dressing, antimicrobial coatings, and detergents. However, despite innumerable applications, the biodegradation of NPs and the effect of their accumulation in the environment remain questionable. The accumulation of bio-degraded NPs within cells leads to intracellular changes, causing alterations in organelle integrity or gene alterations. Although NMs continue to be used extensively, it is important to address their toxicity, environmental impact, and possible undesirable side effects (**Figure 1**). In cancer nanotechnology, monoclonal antibodies, peptides, or small molecules targeting tumor ligands can be induced using NPs to target tumor antigens and tumor vasculature with high specificity and affinity. Recent developments have used bioaffinity NP probes for cellular and molecular imaging to target NP drugs for the treatment of cancers and integrated nanodevices for the early detection of cancers (Gao et al., 2004). Currently, green synthesis of nanoparticles is a novel alternative method. In addition, nanoparticles conjugated with natural biomolecules, which are easily taken up by human cells, have been reported to be more efficacious. In parallel, they act as reducers and stabilizers with smaller sizes and higher penetrating capacities proving themselves to have excellent therapeutic activity.

## Types of green synthesis of nanoparticles

### Biocompatible green reagents

#### Biopolymers

The synthesis of magnetic NPs has attracted a great deal of research interest owing to the utilization of synthetic, non-toxic, and biocompatible materials. The hydrophilic polymer, starch (~20% amylase) plays a substantial role in stabilizing and dispersing NPs, as observed in the synthesis of magnetite NPs (Fe3O4) NPs achieved by sodium alginate biopolymer with a redox-based hydrothermal process using FeCl3·6H2O and urea as a precursor (Jegan et al., 2011).

#### Ascorbic acid functionalizes and stabilizes

NP synthesis. The use of ascorbic acid as a super-paramagnetic iron oxide NPs results in a stable dispersion with an advantage in medical applications. The coated NPs were spherical with a mean particle size of 5 nm, as observed by transmission electron microscopy (TEM) (Sreeja et al., 2014).

#### Amino acids

Biological amines such as L-cysteine, L-arginine, L-glutamine, and L-glutamic acid are functionalized by magnetite NPs to produce FeNPs by a method that involves wet chemical co-precipitation along with the effect of pH (Siskova et al., 2013) (**Table 1**).

**Table 1:** Biosynthetic methods of various kinds of nanoparticles along with their morphology, size, and applications.

Sl. No.	Different types of nanoparticles	Chemical and Biological Agents used	Size/Morphology	Various Applications	References
1.	Bimetallic Fe/Pd-NPs	Starch	14.1 nm	Water purification, degradation of chlorinated hydrocarbons	(He and Zhao, 2005)
2.	Fe3O4	Sodium alginate	27.2 nm/ Spherical	Pollutant removal	(Gao et al., 2008)
3.	Polymer composite-Fe3O4	Agar	50–200 nm/Hexagonal, Spherical	Biosensors, Bio-analysis, gene delivery	(Jegan et al., 2011)
4.	Fe nano-shell	Ascorbic acid (Vitamin C)	<100 nm/cube	Nanomedical field	(Nadagouda and Varma, 2007)
5.	nZVI (Nano zero-valent iron)	Ascorbic acid	20–75 nm/Spherical	Bioremediation of Cd	(Savasari et al., 2015)
6.	Superparamagnetic Iron oxide (Coating and functionalisation	Ascorbic acid	5 nm and 30 nm	Environmental sensing, imaging, and remediation	(Sreeja et al., 2014)
7.	Fe3O4	L-lysine, L-glutamic acid.	17.5 nm/crystalline and spherical	Thermal therapy of cancer	(Vines et al., 2019)
8.	nZVI (Nano zero-valent iron)	L-glutamic acid. L-cysteine L-arginine	–	Bioremediation of Cd	(Siskova et al., 2013)
9.	Fe NPs	Haemoglobin, myoglobin	2–5 nm	Environmental remediation	(Saif et al., 2016)
10.	Fe3O4	Gluconic acid, D-glucose	12.5 nm/Spherical crystalline	Waste water purification	(Lu et al., 2010)
11.	Fe3O4	Gluconic acid, D-glucose	4–16 nm/Crystalline	Drug delivery	(Ramakrishnappa et al., 2022)
12.	Iron NPs encapsulating carbon	Sugars derived from woods	100–150 nm/Nanosphere	To confer extreme chemical stability	(Yan et al., 2015)
13.	Iron oxide	Tannic acid	<10 nm	Drug targeting and Separation of biomolecules	(Herrera-Becerra et al., 2010)
14.	Fe-core shell	Chitosan- Gallic acid	~11 nm/cubic	Molecular bio-imaging and cancer therapy	(Dorniani et al., 2012)

**Figure 2:**
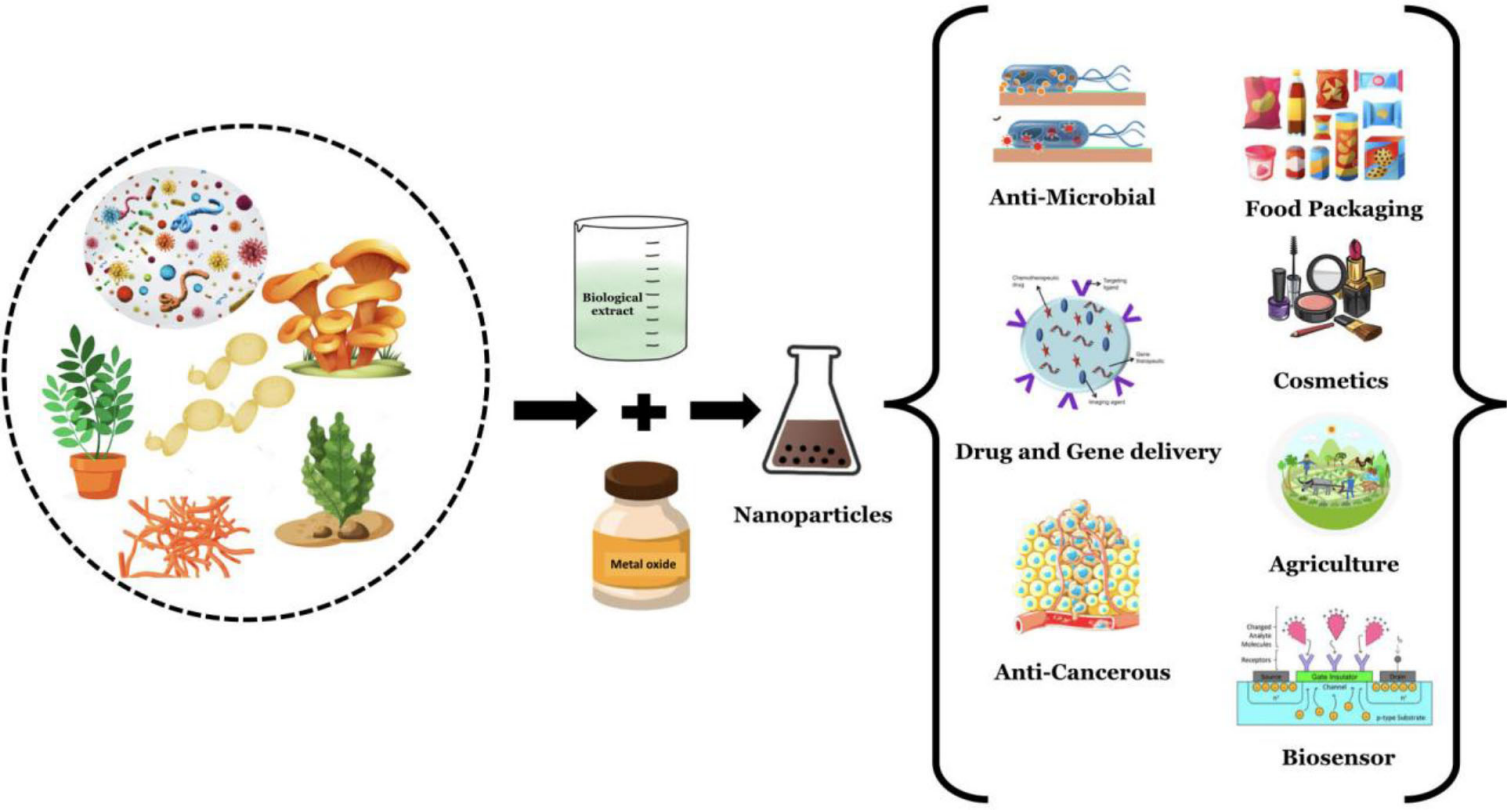
Green synthesis of nanomaterials using various biological components and their applications.

The synthesis of Fe-NPs from natural precursors of Fe, such as hemoglobin and myoglobin, by a single-phase chemical reaction produces Fe-NPs that are stable at room temperature (RT). This strategy is an important approach for the fabrication of bioconjugated NP for biological applications (Sayyad et al., 2012) (**Figure 2**).

#### Sugar and glucose

A reducing agent, D-glucose and gluconic acid as a stabilizer are used for the synthesis of polycrystalline Fe_3_O_4_-NPs (**Table 1**). Synthesis of Fe3O4- NP is coated with glucose and gluconic acid by hydrothermal reduction of Fe^3+^ ions to Fe^2+^ followed by capping of NPs to enhance and stabilize the properties (Ramakrishnappa et al., 2022).

#### Synthetic tannic and gallic acid

Fe_3_O_4_-NPs are exceedingly crystalline and monodispersed and are synthesized by ultrasonication of an aqueous suspension of tannic acid at an optimal pH of 10. These synthesized NPs were spherical with a size less than 10 nm, as revealed by high-resolution transmission electron microscopy (HR-TEM) (Herrera-Becerra et al., 2010). **Figure 2** shows the green synthesis of nanomaterials from different biological agents.

### By microorganisms

#### Bacteria

Iron nanomaterials (Fe-NPs) are biosynthesized most commonly by iron-reducing bacteria, such as *Actinobacteria* sp., under aerobic conditions that form spherical iron oxide nanoparticles (FeO-NPs). These microbes synthesized magnetic NPs when exposed to an aqueous ferric salt solution under aerobic conditions within 48–72 h. The FeO-NPs that exhibited a color change from medium to brown were analyzed by TEM, X-ray diffraction (XRD), Fourier Transform Infrared Spectroscopy (FTIR), magnetic measurements, and other methods. The synthesis of magnetic NPs in bacteria such as *Actinobacter* sp. is a highly complex phenomenon that involves iron reductase enzymes in the presence of excess iron salts (Bharde et al., 2008). Iron reductase reduces Fe^3+^ to Fe^2+^ extracellularly to produce magnetic NPs. Additionally, the activity of Fe^3+^ reductase was confirmed by a ferrisiderophore reductase assay, indicating extracellular synthesis (Bharde et al., 2005) (**Table 2**).

**Table 2:** List of environmental applications involving various microbial-derived NPs.

Sl.No.	Type of Nanoparticles	Type of Microorganisms used	Species Name	Size	Environmental Applications	References
1.	Iron Oxide NP	Bacteria	*Actinobacter* sp. *Bacillus subtilis*	10–40 nm cubic <50 nm 60–80 nm Spherical	–Removal of plant biomass –Microbial enhanced oil recovery, bio-remediation of oil spills	(Bharde et al., 2005; Bharde et al., 2008; Sundaram et al., 2012)
2.	Magnetite NP And Iron NP	Fungi	*Fusarium oxysporum*, *Verticillium* sp. *Aspergillus* *Alternaria alternate*	20–50 nm Spherical 50–200 nm –9 nm	–Fungicides –Visualizing root infections in plants –Antibacterial activity	(Bharde et al., 2006; Pavani and Kumar, 2013; Mohamed et al., 2015)
3.	Iron Oxide NP and Iron NP	Algae	*Sargassum muticum* *Chlorococcum* sp.	18 ± 4 nm, cubic 20–50 nm, spherical	–To treat beach erosion –reduction of chromium	(Mahdavi et al., 2013; Subramaniyam et al., 2015)

#### Fungi

Magnetic NPs are produced extracellularly by a combination of fungi of different sizes, such as *Fusarium oxysporum* and *Verticillium* sp., using ferric and ferrous salts at room temperature. These microbes secrete extracellular cationic proteins that hydrolyze anionic iron complexes to crystalline magnetite particles at low temperatures via means of spontaneous magnetization and ferrimagnetic transition signatures (Bharde et al., 2006). Various species of fungal and bacterial strains, such as *Pochonia chlamydosporium*, *Aspergillus fumigatus*, *A. wentii*, *Curvularia lunata*, *Chaetomium globosum*, *Alcaligenes faecalis*, *Bacillus faecalis*, and *Bacillus coagulans*, have been tested to produce iron NP (Kaul et al., 2012) (**Table 2**).

#### Algae

The biosynthesis of Fe_3_O_4-_ NP involves ferric chloride reduction in macroalgae of brown seaweed, *Sargassum muticum* extracts containing sulfated polysaccharides that help facilitate the reduction of iron salt. XRD analysis of the NPs synthesized using this method indicated the presence of crystalline and cube-shaped particles (**Table 2**). The TEM images of these microalgae revealed the presence of intracellular and extracellular iron. FTIR analysis confirmed that carbonyl and amine groups reduced the iron salts (Mahdavi et al., 2013). Algae are potential phototrophic organisms known for the greater production of several metabolites, pigments, polysaccharides, fibers, and secondary metabolites, yielding larger production of NPs. Hence, these biofactories are expected to contribute more competent and harmless bioactive compounds for various applications.

### Nanoparticles synthesized by plants

Green synthesis of metallic NPs from various plant parts, such as leaves, stems, roots, and seeds, is the simple, inexpensive, and reproducible. However, plants as natural sources will certainly produce the added advantage of stable metal NPs. Hence, green-synthesized NPs have proven to be the best candidates for fast and large-scale production in comparison to microbes that require specific environmental conditions (Kalaiarasi et al., 2010). Extracts from the seeds, fruits, leaves, stems of different herbs and higher plants are rich in antioxidants that help boost the capacity of NPs. Therefore, the use of plant-based phytochemicals for the synthesis of NPs unites natural/plant sciences and nanotechnology, and enables safe, environmentally friendly, justifiable, and economically feasible green technologies (Mayegowda et al., 2022a; Mayegowda et al., 2023).

#### Synthesis from leaf extract

Fe-NPs were produced using green tea (*Camellia sinensis*) extracts, which are rich in polyphenols. Polyphenols are excellent reducing and capping agents, resulting in the formation of stable, green, nanoscale zero-valent iron particles (Rotti et al., 2023). Similarly, crystalline monodisperse magnetite [Fe_3_O_4_] NPs were synthesized using the carob leaf in a one-step reaction (Awwad and Salem, 2012). FeO/Fe_3_O_4_ NPs were synthesized using pomegranate (*Punica granatum*) leaf extract and heat-killed yeast (*Yarrowia lipolytica*), as biosorbents to remove hexavalent chromium. Mössbauer spectroscopy revealed the presence of FeO/Fe_3_O_4_, and SEM images displayed even the dispersal of Fe-NPs on the outer surface of yeast cells (Rao et al., 2013).

#### By fruit extract

Pd and Fe-NPs were prepared using aqueous fruit extracts of *Terminalia chebula.* Stable Fe-NPs were synthesized by the reduction of a FeSO_4_·7H_2_O solution by *T*. *chebula* extract (Kumar et al., 2013). Fe_3_O_4_ NPs were synthesized from the fruit extracts of *Passiflora tripartita* var. *mollissima* are 22.3 nm in size and show significant nanocatalytic activity (Kumar et al., 2014).

#### Seed extract

A previous study demonstrated the production of FeO NPs using *Syzygium cumini* (Malabar plum) as a stabilizing agent. The seed extract was used as a reducing agent, and sodium acetate was used as an electrostatic stabilizing agent. The presence of polyphenols, such as flavonoids, was confirmed by FTIR spectroscopy, which is important in serving the role as reducing agents (Venkateswarlu et al., 2014).

## Mechanism of antimicrobial action

### Bacteria

**Figure 3:**
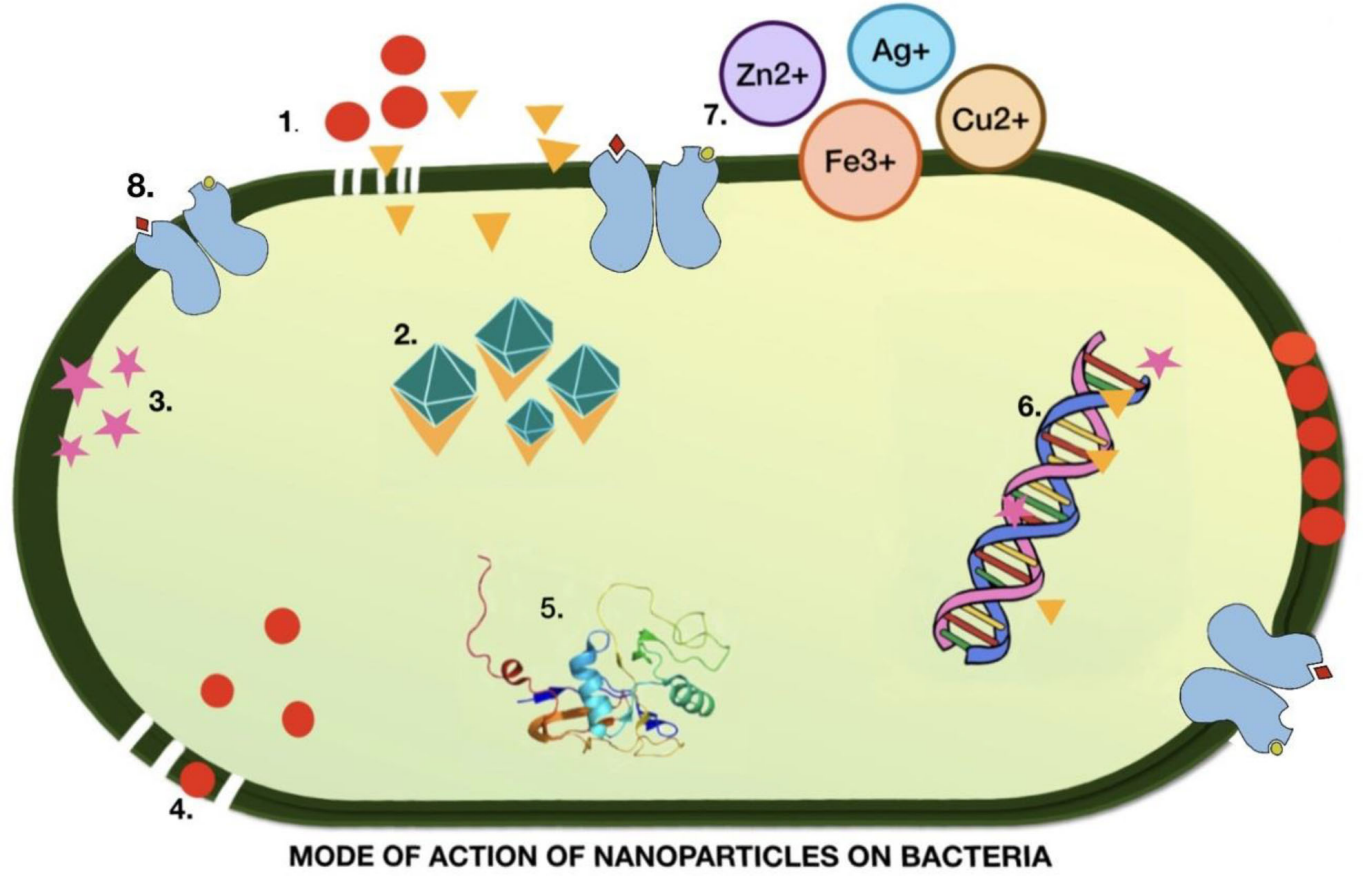
Illustration of the mechanism of action of nanoparticles in the destruction of bacterial cells. 1. Metal nanoparticles destroyed the cell wall and cell membrane. 2. Ions generated from metal NP bind to and denature ribosomes. 3. ROS production. 4. Accumulation of nanoparticles rupturing the cell membrane 5. Alteration of the protein structure leading to damage. 6. DNA denaturation. 7. Metal ions that inhibit the electron transport chain. 8. Metal NPs bind to the receptor, causing a change in confirmation.

NPs also exhibit antibacterial properties. Silver (Ag) NPs continuously release Ag ions that adhere to the bacterial cell wall and cell membrane, leading to disruption of the bacterial envelope (Khorrami et al., 2018). Once inside the cell, Ag deactivates respiratory enzymes by producing reactive oxygen species (ROS) (Ramkumar et al., 2017). The accumulation of ROS causes damage to DNA, RNA, and proteins, resulting in cell death. The Ag released from NPs binds the sulfur and phosphorus of DNA and blocks replication and cell division, eventually leading to cell death. Ag ions also cause denaturation of ribosomes in the cytoplasm, leading to inhibition of protein synthesis (Durán et al., 2016; Jadimurthy et al., 2022) (**Figure 3**).

The accumulation of AgNPs alters the structure of the cell membrane and promotes cell lysis (Liao et al., 2019). The surface charge of the NP plays a significant role in determining its interaction with the cell membrane. Cationic NPs infiltrate cells and cause extensive damage. Although anionic NPs do not penetrate the plasma membrane, they destabilize at specific concentrations. While this property of charged NPs suggests potential damage to human cells, it offers opportunities for use as a vehicle for drug delivery to cancerous cells. Denaturation of the cytoplasmic membrane often leads to the rupture of organelles, which ultimately results in cell lysis. Bacterial signal transduction involves phosphorylation events that enable bacteria to sense, adapt, and respond to environmental changes. NPs cause dephosphorylation and disruption of signal transduction, leading to apoptosis (Li et al., 2019). Ag NPs are spherical or quasi spherical and can easily release Ag ions owing to their large surface (Shanmuganathan et al., 2018). To avoid agglomeration, capping agents are used to coat the NPs, which modifies their surfaces and affects their dissolution. The presence of organic or inorganic in the medium can also be responsible for the dissolution of NPs by aggregation.

It has been demonstrated that Ag NPs release Ag ions in the aqueous solution and this is quite faster when the pH is acidic (below 5) than in neutral solution (Jacob et al., 2019). Differential mode of action for NPs has been reported for Gram-positive and Gram-negative bacteria and has been attributed to the presence of peptidoglycan in Gram-positive bacteria which hinders efficient penetration (Meikle et al., 2020). NPs with a size less than 10 nm can directly penetrate the cell and alter cell permeability, enter bacteria, and cause cellular destruction, suggesting a direct correlation between NP uptake and antibacterial properties (Noronha et al., 2017). Hence, Ag NPs have a great potential as antibacterial agents. However, the application of Ag NPs as biofilm inhibitors warrants further investigation (Pugazhendhi et al., 2018). Magnesium oxide NPs have been reported to possess anti-biofilm and anti-adhesion potential, which are efficient against drug-resistant bacteria (Hayat et al., 2018).

### Fungi

Repetitive damage to the environment due to hazardous chemicals, such as excessive use of pesticides, poses a great threat to agriculture worldwide. The use of NPs to target plant pathogens appears to be a safe alternative to chemically synthesized pesticides (Kim et al., 2012). Ag NPs, with varied modes of action, are suggested to be safe agents against plant pathogens, particularly commercially relevant fungal pathogens, as compared to artificial antifungal agent (Manjula et al., 2023). Metal NPs are non-toxic, eco-friendly, and widely used as disinfectants and investigational antifungal mediators (Borehalli Mayegowda et al., 2022). Zinc NPs have potential applications in the pharmaceutical sector, as potent antimicrobial agents and in water disinfection. Studies demonstrating the antifungal activity of Ag NPs are limited. Kim and colleagues have however, suggested a concentration dependent inhibitory activity, probably due to saturation of fungal hyphae with higher density NPs leading to deactivation of the disease-causing fungi (Kim et al., 2012).

While the effect of Ag ions on fungi is limited, reports suggest their inhibitory effect on DNA replication (Feng et al., 2000) and impairment of ribosomal attachment, blocking protein synthesis, enzymes, and additional proteins involved in the production of ATP (Yamanaka et al., 2005). Ag NPs synthesized using ribose sugar and sodium dodecyl sulfate acting like reducing agent and capping agent, respectively demonstrating antifungal activity against highly resistant human pathogenic fungi such as *Candida albicans* and *Candida tropicalis* (Mallmann et al., 2015). Similar results have been reported by other researchers (Kim et al., 2009). Fungal cells maintain an ion gradient, trehalose and glucose protects the biological viability of cells from protein denaturation caused by environmental stress such as heat, cold, high pH, dehydration, oxidative stress, and lethal agents (Alvarez-Peral et al., 2002). NPs disrupt the membrane structure and permeability, leading to leakage of intracellular contents and loss of membrane potential. TEM observations revealed the formation of pits in the fungal membrane when treated with Ag ions, leading to cell cycle arrest and cell death in *C. albicans*. Additionally, destruction of the membrane and inhibition of budding have been noted (Endo et al., 1997). Palladium NPs demonstrated effective antifungal activity against *Colletotrichum gloeosporioides* and *F. oxysporum* although in a size-dependent manner (Osonga et al., 2020). Therefore, NPs may induce antifungal activity by disrupting cellular integrity, generating reactive species, and creating osmotic imbalances in pathogens.

### Virus

While several viral diseases have been eradicated, emerging threats from novel viruses cannot be ignored because of their adaptability and mutagenic ability (Esteban, 2010). Hence, these viruses pose a continuous challenge to the scientific community. Enfuvirtide is a synthetic peptide drug, approved by the US Food and Drug Administration that targets a specific HIV protein named the gp41 coding envelope protein and prevents its fusion (Galdiero et al., 2011).

**Figure 4:**
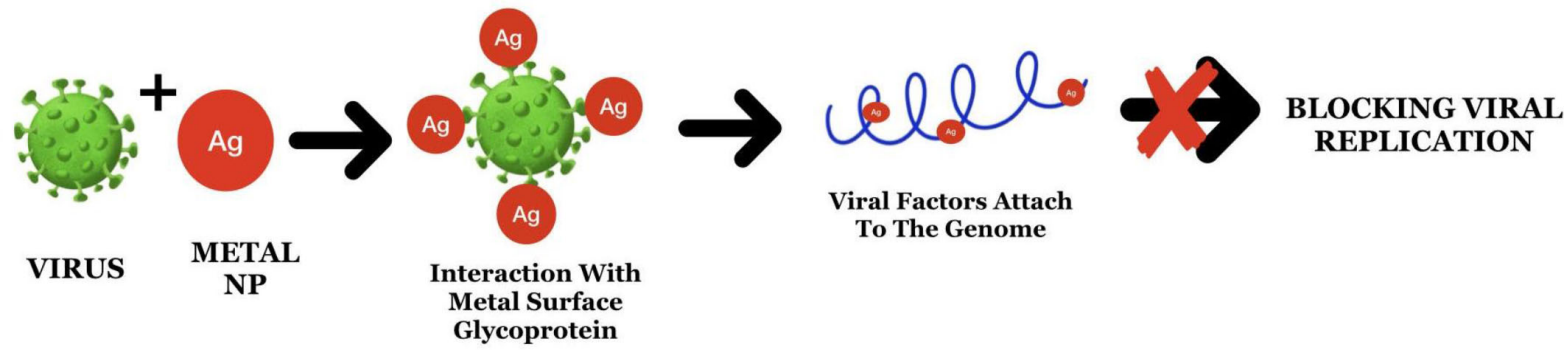
The mode of action of Ag nanoparticles through attachment to the surface proteins and viral genome leads to the blockage of replication.

Under such challenging circumstances, NPs have been developed as promising antiviral agents owing to their increased surface area and unique chemical and physical properties (Elechiguerra et al., 2005). NPs block viral infections by having their mode of action at the time of attachment as well as entry by obstructing polyvalent interactions across viral surface components in interaction with host cell membrane receptors (**Figure 4**) (Helenius, 2007). Previous studies have demonstrated the efficacy of NPs against HIV-1 (Lara et al., 2010a; Lara et al., 2010b), hepatitis B virus (Lu et al., 2008), herpes simplex virus type 1 (Baram-Pinto et al., 2009), monkey pox virus (Rogers et al., 2008), and influenza virus (Papp et al., 2010). While the interaction and efficacy of NPs against viruses were largely dependent on NP size, they also interacted at specific sites. NPs also bind strongly to the sulfur-containing residues of GP120 (Lara et al., 2010a). Both Ag and Au NPs have promising antiviral properties, particularly against enveloped DNA/RNA viruses. A recent study on COVID-19 viral disease explains about the neurotropism property demonstrating the application of NPs by inhibiting replication by cellular transcytosis blockade when given in the form of encapuslted particles (Ren et al., 2021).

## Characterization of nanoparticles

NPs are classified based on their composition into three classes: a) organic-based, which comprises proteins, lipids, carbohydrates, polymers, or any other organic compounds that are nontoxic, ecofriendly, such as dendrimers, liposomes, micelles, and protein complexes (Bharde et al., 2005; Bharde et al., 2008; Rotti et al., 2023), and carbon-based are solely made of carbon such as fullerenes, carbon black NPs, and quantum dots (Savasari et al., 2015). Inorganic nanoparticles (NPs) including metals, ceramics, and semiconductors. These inorganic metal NPs can be synthesized from monometallic or bimetallic alloys (Mayegowda et al., 2022b). Owing to the varying properties of NPs, different techniques can be used for characterization and analysis to determine their potential applications. Morphological and topological features were established to check the size, shape, dispersity, localized, surface topography, surface area and porosity of NPs, which0 can be determined by TEM and SEM (Herrera-Becerra et al., 2010; Sreeja et al., 2014). Other techniques include dynamic light scattering (DLS) and nanoparticle tracking analysis (NTA), which involve the light interference measured on the suspended NPs for Brownian motion with the correlation of its velocity with their size using the Stokes–Einstein equation (Bharde et al., 2008). However, the structural and chemical characterization of NPs can be carried out by XRD, energy-dispersive X-ray spectroscopy (EDX), high-angle annular dark-field imaging (HAADF), and FTIR for the phase, composition, crystallinity, chemical state (oxidation), functional groups, surface charges, and electrochemical characteristics. Zeta potential analysis can be used to determine the surface charge of NPs (Manjula et al., 2023; Mayegowda et al., 2023). The characterization of NPs for optical, electronic, and electrical properties was carried out by Raman spectroscopy and SERS for absorption, luminescence, electronic state, photoactivity, and electrical conductivities. UV–vis and photoluminescence spectroscopy, which transitions electrons from the ground to an excited state, as measured by absorption spectroscopy (Manjula et al., 2023). Magnetic properties have great advantages and are quantified by magnetic force microscopy or electron spin resonance spectroscopy.

## Application of nanoparticles

Although NPs hold tremendous promise owing to their ease of synthesis and wide industrial applications, their synthesis using chemical approaches results in toxic side effects. Therefore, green synthesis is promising as an alternative and safe process. The following section highlights the commercial, medicinal, and other applications of green synthetic NPs.

### Agriculture

In agriculture, nanotechnology is primarily used for the synthesis of pesticides and fertilizers. NPs can be used as fertilizers to facilitate crop improvement, seed germination, and root growth, while also reducing eco-toxicity. Ag NPs were shown to improve wheat growth and yield. One of the essential micronutrients for plant metabolic processes is zinc, which is involved in the synthesis of auxins such as indole acetic acid and carbohydrates, and in the formation of chlorophyll. Many NPs have been used in the form of pesticides, such as green peach aphids (Ghidan et al., 2018; Mayegowda et al., 2022b). Decreased plant mortality for granulomatosis with polyangiitis (GPA), a condition in which the blood vessels become swollen, was achieved by the synthesis of NPs using aqueous peel extracts of *Punica granatum*, leaf extracts of *Olea europaea*, and flower extract of *Chamaemelum nobile*. GPA infection and the absence of metal accumulation suggest the advantages of bio-NPs (Ghidan et al., 2017). Ag NPs also function as fungicides and have been effective against *C. gloeosporioides* which causes bitter rot in various crops (Aguilar-Méndez et al., 2010).

### Food industry

Nanotechnology in the food industry can escalate their shelf life of various foods and reduce their depletion due to contagious infestation (Pradhan et al., 2015). Encapsulation of biomolecules, such as lipids proteins, carbohydrates, and vitamins, using NPs can protect them from the acidic environment of the stomach and intestine and can improve their assimilation (Singh et al., 2017). However, the use of NPs in food packaging materials aids in the preservation and protection from several of these issues.

Essential oils obtained from organic compounds, organic acids, and bacteriocins have been extensively studied for use in polymeric matrices as antimicrobial packaging material (Gálvez et al., 2007). Many NPs, such as Ag, copper, biopolymer chitosan, and metal-oxide derived NPs, such as titanium oxide (TiO_2_) and zinc oxide (ZnO), have been shown to have antibacterial activity (Manjula et al., 2022). Nanobiosensors are used in the food industry for pathogen detection during processing (Mayegowda et al., 2022). Nano-biosensor can detect changes in the environment, such as moisture content or temperature fluctuations in storage rooms, microbial contamination, or product deprivation. NPs play a vital a role in the food industry, and their green synthesis offers additional advantages.

### Cosmetics

The use of NPs is most popular in the cosmetic industry. Leading brands such as L’Oreal, Avon, and Johnson and Johnson have patents on nanoparticles. NPs are employed in sunscreens, skin creams, skin lotions, and dye-based products such as fabric colors, skin tanning, and whitening lotions. Sunscreens are used as UV filters and provide broad UV protection with minimal or no adverse effects. NMs labeled in cosmetics can be similar to vesicles in cells, such as liposomes, nano-emulsions, and nano-capsules. Other solid lipid NP conjugates, include nanocrystals and NP with metal-like nanosilver and nanogold, dendrimers, cubosomes, liquid-based hydrogels, and buckyballs (Raj et al., 2012; Ananda et al., 2022). NPs utilized in cosmetics are generally chemically synthesized, but there is an emerging shift towards green synthesis. Xia et al. demonstrated the use of ivy plant-derived NPs as an alternative to oxide particles in blocking UV rays (Bezbaruah et al., 2022).

### Healthcare

Recently, NPs have been used for detection, diagnosis, and tumor therapy. NPs are excellent tools for cancer drug delivery because of their small size, which facilitates targeted delivery. NPs have also been used to mitigate adverse effects of photodynamic cancer therapy. The hydrophilic antigenic drug Mosqurix^®^ is used to treat malaria caused by *Plasmodium falciparum* and hepatitis B virus. This liposomal drug, modified as a phospholipid intrinsic adjuvant, has greater stability in the gastrointestinal tract for absorption. A nanogel made up of polyethylenimine along with cationic coated alginate, which is given intraperitonially, produces anti-ovalbumin IgG, which is promising for drug delivery (Adarsha et al., 2022). A dye used in photodynamic therapy migrates to the skin and eyes, leading to sensitivity. Partial encapsulation of the dye in NPs helped reduce sensitivity (Roy et al., 2003). Furthermore, NPs, such as gold NPs, have research applications, such as immunohistochemistry and identification of protein–protein interactions. Cao et al. (2003) synthesized a AuNP probe with catalytic activity for protein identification (Cao et al., 2003; Yadav et al., 2021). The extensive medical use of chemically made NPs has been reported to have adverse effects. Therefore, there has been a shift toward safer green synthetic bio-NPs. A study reported the use of nano-encapsulated AgNPs by green-synthesized pcDNA3.1/H5 for immunization through humoral and cell-mediated immune responses. Thus, green NPs can be used as an alternative DNA vaccine through nanotechnology for the oral administration of vaccines in a more effective way. AgNPs are an attractive and unique alternative delivery method for oral DNA vaccinations. NP-based vaccination showed a similar effect in the elucidation of the immune response as traditional vaccination procedures. In addition, it exhibits greater stability, cost-effectiveness, and target specificity in minimizing various disease types (Jazayeri et al., 2012).

Fungal based synthesized ZnNPs showed excellent antimicrobial property against pathogenic fish bacteria in comparison with chemical ZnNPs. This study also highlights the increase in immune response in organisms by the released Zn^2+^ hindering active transport as well as inhibiting bacterial enzymes in metabolism, leading to the release of ROS, causing death of the organism (El-Saadony et al., 2021). Similarly, biosynthesis of NPs from microbes and plant extracts has also shown vast application in modulating immune responses through innate and adaptive immunity across conditions, such as inflammation, cell differentiation and signaling during cancer, autoimmune disease, and allergic reactions. Hence, describing the ecofriendly and safer approach to green NPs is a novel therapeutic approach in drug delivery and therapeutics (Cai et al., 2022).

## Toxicity of nanoparticles

Despite the innumerable advantages of NPs, as listed above, they can still be toxic at higher concentrations, if overused, or accumulate within the system. However, the toxicity levels in the environment depend largely on the type of NPs and the properties that have been used.

### Toxicity to humans

**Figure 5:**
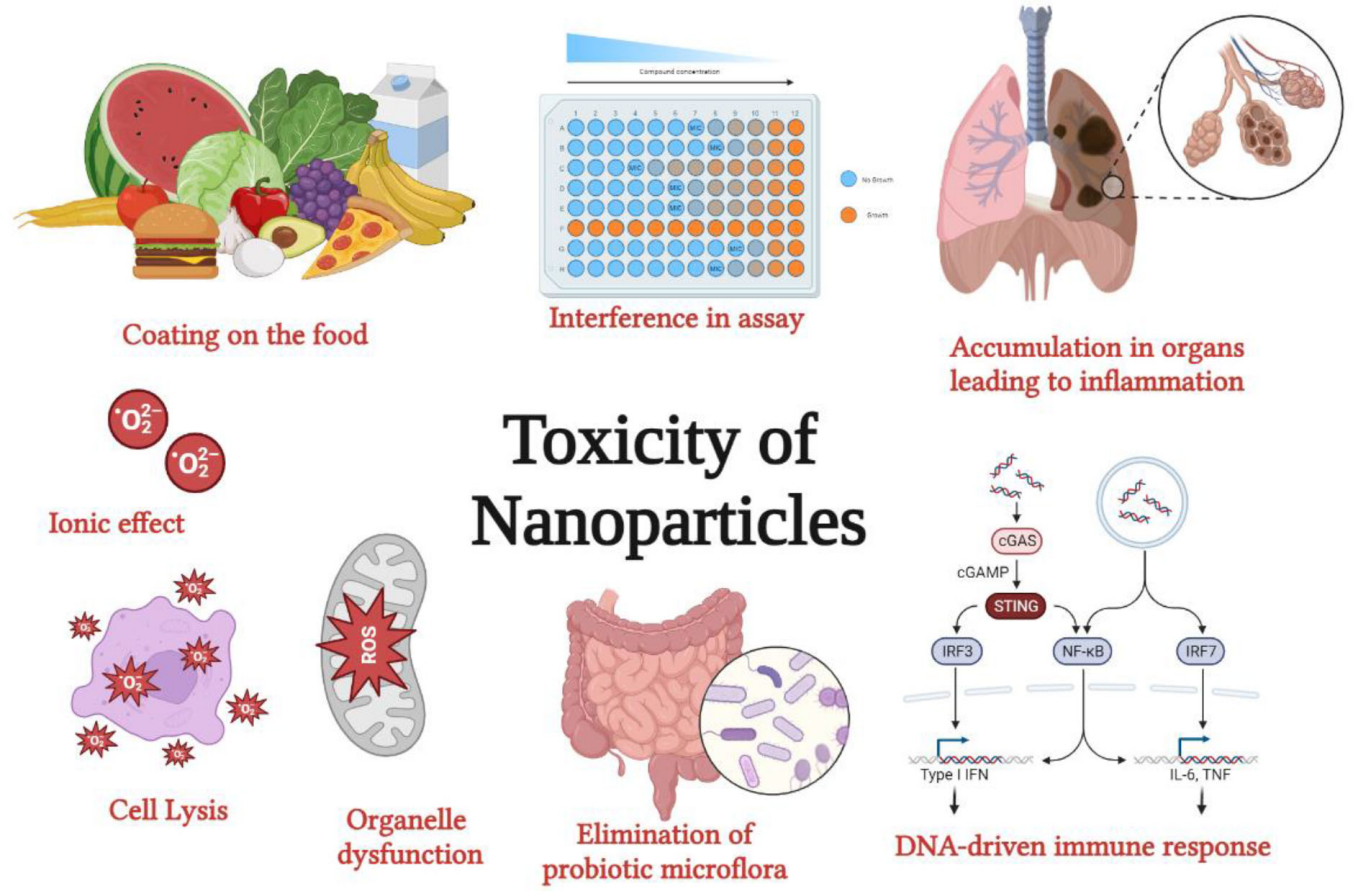
Side-effects of overdosing of nanoparticles on different levels of organization.

The size of the NP decreased from 30 to 3 nm, and the number of surface molecules increased from 10% to 50%. Metal NPs are the most common type of NPs employed in all industries. Studies on cellular uptake of gold NPs indicated their extracellular aggregation followed by enmass transport into the cells with resultant reactive species production and cytotoxicity (**Figure 5**) (Goodman et al., 2004; Chithrani et al., 2006). Studies have shown that the formation of abnormal actin filaments results in decreased cell adhesion, proliferation, and motility (Pernodet et al., 2006). While gold nanospheres capped with citrate were non-toxic to baby hamster kidney and human hepatocellular liver carcinoma cells, they were toxic to human carcinoma lung cell lines at certain concentrations, signifying their effect on cell type (Pernodet et al., 2006).

Large concentrations of Ag in atmospheric air led to breathing abnormalities and lung, throat, and stomach discomforts. Ag n skin contact can occasionally lead to insignificant allergic conditions, such as rashes, swelling, and inflammation. However, several studies have been conducted (Carlson et al., 2008). However, studies have not proven the toxicity of spherical AgNPs and Ag nano-prisms in human skin keratinocytes. TiO_2_ NPs induce oxidative stress by inducing changes in gene expression (Park et al., 2008). ZnO also induced toxicity by generating reactive oxygen species, oxidant injury, and inflammation, leading to cell death (El-Saadony et al., 2021).

### Toxicity to the environment

In addition to humans, other animals and microbes such as zebra fish, daphnids, and algal species were exposed to silver, copper, aluminum, nickel, cobalt, and TiO_2_ nanoparticles. Griffitt et al. (2008) detected toxicity levels in aquatic organisms owing to the presence of metallic NPs. In contrast, no toxicity was observed for TiO_2_ NPs. These results were corroborated by another study on zebrafish embryos that suggested the dose-dependent developmental toxicity of Ag NPs (Asharani et al., 2008). The toxicity level of AgNPs in the aquatic environment can be denoted by the concentration accumulated by aquatic organisms. The liver is the initial accumulator organ for NPs in these aquatic organisms, followed by the gills, intestine, and muscles, causing cell necrosis and lysis in the intestinal villi, followed by progressive release of Ag ions (Liu et al., 2015). AgNPs are frequently used in apparel owing to their antimicrobial properties. However, the release of Ag, in both colloidal and ionic forms, from the fabric during washing causes its accumulation in the wastewater, leading to toxicity (Benn and Westerhoff, 2008).

ZnO NPs are readily used in cosmetics and, as such, are a large constituent of factory run off from the cosmetic industries. These NPs are toxic to *Lolium perenne* (ryegrass). ZnO-NP, along with the ryegrass biomass, has been able to suggestively reduce, characterized by shrinking the root tips, and highly vacuolated or collapsed epidermal or cortical cells (Lin and Xing, 2008). However, a minor reduction in root cells was detected upon exposure to uncoated alumina NPs (Yang and Watts, 2005).

## Future prospects

Recently, there has been a burst of commercial applications for NPs. However, green synthesis of NPs is still in its infancy. Green synthesis offers a safe mechanism for producing nontoxic NPs with additional beneficial effects. Green synthesis has already been applied in several fields owing to the use of natural alternatives. Bio-NPs have been used as artificial food colors, strengthening agents for skincare products, antimicrobial agents embedded in fabrics, core molecules for drugs, and therapeutic molecules. However, their global application is limited by technology. Further research in this nascent field is warranted. Thus, green synthesis has the potential to reduce environmental pollution by employing commercially viable yet safe biomaterials.

## Conclusion

NPs are generally used for their antimicrobial properties, which can act against bacteria, fungi, and certain viruses. This generally results from the metal component, but bio-NPs also contain other biomolecules as vital components. The antimicrobial properties of NPs are used in various industries, such as food packaging, active agents in skincare products, disease treatment, and drug delivery. It should be noted that the overuse and extensive deployment of NPs could lead to toxicity owing to the accumulation of metals and ions being released. However, lethal effects on humans at the currently used concentrations have not been reported. In conclusion, green synthesis is a largely positive and significant venture in all fields of science, in which the use of environmentally friendly resources and biodegradable materials in the synthesis of NPs will undoubtedly lead to an eco-friendly era with reduced industrial and environmental pollution.

## Author contributions

SB, AR, and MG conceptualized, designed, and wrote the initial manuscript draft the manuscript. SP, SA, MAlm, MAll, NSA and RS prepared the figures and tables, edited, and revised the manuscript critically. Final manuscript has been approved by all the authors.
